# Evaluation and integration of existing methods for computational prediction of allergens

**DOI:** 10.1186/1471-2105-14-S4-S1

**Published:** 2013-03-08

**Authors:** Jing Wang, Yabin Yu, Yunan Zhao, Dabing Zhang, Jing Li

**Affiliations:** 1 Bor Luh Food Safety Center, National Center for Molecular Characterization of Genetically Modified Organisms, State Key Laboratory of Hybrid Rice, School of Life Science and Biotechnology, Shanghai Jiao Tong University, China; 2 Department of Bioinformatics & Biostatistics, School of Life Science and Biotechnology, Shanghai Jiao Tong University, China; 3 School of Medicine, Shanghai Jiao Tong University, China; 4 School of Electronic Information and Electrical Engineering, Shanghai Jiao Tong University, China; 5 Shanghai Center for Bioinformation Technology, China

## Abstract

**Background:**

Allergy involves a series of complex reactions and factors that contribute to the development of the disease and triggering of the symptoms, including rhinitis, asthma, atopic eczema, skin sensitivity, even acute and fatal anaphylactic shock. Prediction and evaluation of the potential allergenicity is of importance for safety evaluation of foods and other environment factors. Although several computational approaches for assessing the potential allergenicity of proteins have been developed, their performance and relative merits and shortcomings have not been compared systematically.

**Results:**

To evaluate and improve the existing methods for allergen prediction, we collected an up-to-date definitive dataset consisting of 989 known allergens and massive putative non-allergens. The three most widely used allergen computational prediction approaches including sequence-, motif- and SVM-based (Support Vector Machine) methods were systematically compared using the defined parameters and we found that SVM-based method outperformed the other two methods with higher accuracy and specificity. The sequence-based method with the criteria defined by FAO/WHO (FAO: Food and Agriculture Organization of the United Nations; WHO: World Health Organization) has higher sensitivity of over 98%, but having a low specificity. The advantage of motif-based method is the ability to visualize the key motif within the allergen. Notably, the performances of the sequence-based method defined by FAO/WHO and motif eliciting strategy could be improved by the optimization of parameters. To facilitate the allergen prediction, we integrated these three methods in a web-based application proAP, which provides the global search of the known allergens and a powerful tool for allergen predication. Flexible parameter setting and batch prediction were also implemented. The proAP can be accessed at http://gmobl.sjtu.edu.cn/proAP/main.html.

**Conclusions:**

This study comprehensively evaluated sequence-, motif- and SVM-based computational prediction approaches for allergens and optimized their parameters to obtain better performance. These findings may provide helpful guidance for the researchers in allergen-prediction. Furthermore, we integrated these methods into a web application proAP, greatly facilitating users to do customizable allergen search and prediction.

## Background

Allergy and other hypersensitivity reactions from the foods and environmental factors are major causes of chronic ill health in the world [1,2], affecting about 25% of the population [3,4]. Allergens include proteins in food, cold air, hot air, ultraviolet rays, metal, and so on. Among these allergenic proteins may cause possible great dangers to health. Therefore, assessment of the potential allergenicity of proteins is essential for food production.

Over the last 15 years, several documents have been officially released providing guidance for definition of the potential allergenic proteins [5-7]. ILSI (International Life Sciences Institute) Allergy and Immunology Institute provided a science-based decision tree approach to assess the allergenic concerns associated with the introduction of gene products into new plant varieties in 1996. Codex Alimentarious Commission advanced the 'decision tree' twice in 2001 and 2003 to achieve a better performance. DuPont Experimental Station presented a "weight-of-evidence" approach, which take into account a variety of factors and approaches for an overall assessment of allergenic potential [7,8]. This guideline suggested the assessment ranging from the source of novel proteins, similarities of the target proteins to known allergens at the primary protein sequence level, the physicochemical properties, and protein abundance etc.

To enforce the requirement of evaluation of allergenicity of novel proteins, several computational approaches have been developed for effectively screening the possible allergenicity of proteins. The first computational approach proposed by the consultation group of FAO/WHO in 2001, defined a possible allergenic protein with the exact match a stretch of six or more consecutive identical amino acids (rule 1) or more than 35% identity within any window of 80 amino acids in comparison with any known allergen (rule 2) [6]. This sequence-based approach has been widely accepted for allergen prediction using web tools, such as Allermatch, AllerTool and AllergenPro [9-11]. However, it was reported that only 1 of 200 "positive matches" represents a true allergen when using FAO/WHO guidelines in 2003[12]. Subsequently, a motif-based approach using the secondary structure of proteins was proposed for allergen prediction with an increase of the precision from 37.6% to 94.8%, while its recall decreased from 97.0% to 86.2% [12]. In 2006, a statistical learning method SVM (support vector machine) was developed using the principle of pattern recognition [13-17]. Furthermore, additional two approaches: epitope- and ARPs-based (Allergen Representative Peptides) methods were reported using common subsequences of target proteins [13-20]. These two methods were limited by few known epitopes and allergenic domains.

Although a variety of computational methods for allergen prediction have been reported, there exists no comprehensive comparison of these methods. Motif-, epitope-, ARPs- and SVM-based approaches were attempted to be compared in the previous study [13], but the sequence-based method was not included and only one motif for one subset was selected for prediction, which may cause prediction with low sensitivity. In this article, we comprehensively evaluated the performances of sequence-based, motif-based and SVM-based allergen prediction approaches using the training and testing datasets respectively. Further, these approaches were integrated and optimized in a web-based application proAP to provide a comprehensive, integrative and friendly resource for allergen prediction.

## Methods and materials

### Data set

**Figure 1:**
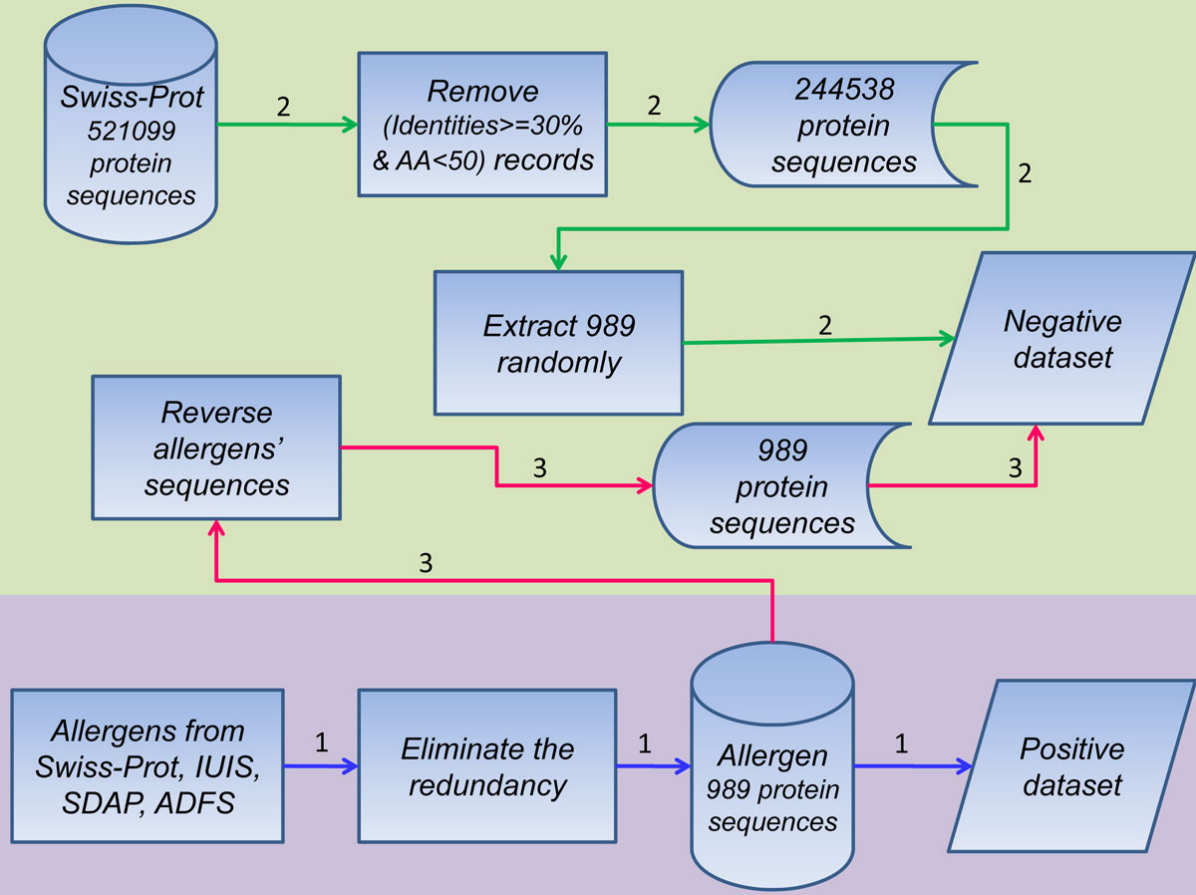
**The flow diagram of dataset collection**. The setup processes of the positive dataset were displayed in blue lines and that of the negative dataset were in green. And a reversed negative dataset was built as the flow in dark pink for evaluating FAO/WHO rule 2 specifically.

The allergens were obtained from various sources including (1) Swiss-Prot Allergen Index: http://www.uniprot.org/docs/allergen.txt, (2) IUIS Allergen Nomenclature: ihttp://www.allergen.org/, (3) SDAP: http://fermi.utmb.edu/SDAP/, (4) ADFS: http://allergen.nihs.go.jp/ADFS/index.jsp. We got 989 allergen protein sequences in total after integrating the data and removing redundant ones which have more than 99% similarity only within the same species. These 989 allergens aforementioned were used as the positive dataset (allergens) which are originated from 249 distinct species. To build a reliable negative dataset, we downloaded 522,019 protein entries from Swiss-Prot (Swiss-Prot Release 2010_11 of 02-Nov-10), then removed the entries of which identities> = 30% with any known allergen and removed the proteins of which sequence length<50. Finally the remaining 244,538 records can be sampled randomly as negative controls (presumptive non-allergens). Since we removed the entries with sequence identities> = 30% with any known allergen, the setup of negative dataset may raise the risk of over fitting when the assessment was performed according to the method described by FAO/WHO rule2. To avoid this risk, as the way adopted by Stadler et al. [12], the reversed sequences of all allergens were taken as negative dataset for the evaluation of FAO/WHO rule 2. The flow diagram of dataset collection was summarized in Figure 1.

### Methods for allergen prediction

#### Sequence-based approach

As mentioned above, sequence-based approach was proposed by FAO/WHO [7], which required doing amino acid sequence similarity analysis in comparison with known allergens. *Wordmatch *programming by *Perl *was developed to meet the requirement of FAO/WHO rule 1 [21], and this method searches short sub-sequences (*words*), which have perfect identity with an allergen entry [9]. To implement rule 2, the query protein sequence was divided into 80 amino acids by a *sliding window *with steps of a single residue, then each of these windows used to align to all allergen sequences using *blast-2.2.23 *[22]. The *wordsize *(the number of consecutive identical amino acids exactly matched) and the identity threshold were set to be configurable.

### Motif eliciting strategy

Unlike sequence-based approach, motif-based approach relies on the protein secondary structure (motif) instead of primary structure (amino acid sequence). The motif-based approach included the extraction of the characteristic motifs from known allergens and subsequent comparison of the query proteins with these motifs. Generally it starts with the positive dataset, then the following steps were performed iteratively until no motif with E-value less than 0.01 was found: the most relevant motif contained in the allergen sequences was identified using MEME motif discovery tool [23]; the generalized profile of the identified motif was scaled on the allergens with MAST [24]; matching allergen sequences were removed from the allergen database, and remaining sequences were submitted to the next iteration of motif discovery.

### Feature vectors computation in SVM-based prediction

SVM (Support vector machine) is a statistical learning method, which performs classification by constructing an N-dimensional hyperplane that optimally separates the data into two categories. For allergens prediction, take the features of the known allergens and the non-allergens as input to SVM for modeling, and then SVM predicts the query as allergen or non-allergen according to the model.

In this study, the SVM has been implemented using LIBSVM software [25]. As reported by Saha *et al. *[13], the input vectors we selected were the most commonly used amino acid composition in SVM-based predict approach. Amino acid composition is the fraction of each amino acid in a protein. The fraction of all 20 natural amino acids was calculated using the Eq. (1).

(1) $$\text{Fraction of amino acid }i=\;\frac{\text{total number of amino acids (}i\text{)}}{\text{total number of amino acids in protein}}$$

, where  i can be any amino acid. And then these compositions were utilized as input vectors of dimension 20 for testing.

### Tenfold cross-validation

The performances of all computational methods applied in this study were evaluated using ten-fold cross-validation. The dataset was randomly partitioned into ten subsets, where each subset had nearly equal number of allergens and non-allergens. Of the ten subsets, a single set was retained as the validation data for testing the method, and the remaining nine subsets were used as training data. This process was then repeated 10 times with each of the ten subsets used exactly once as the validation data. The overall performance of a method was the average performance over ten subsets.

### Performance measurements

Several statistics measurements were used to evaluate the performance of each allergen prediction methods presented in this study and were briefly described as below [26]:

**• Sensitivity**, also referred to as recall, is the percentage of correctly predicted allergens. It is derived by the Eq. (2).

**• Specificity **is the percentage of correctly predicted non-allergens. It is derived by Eq. (3).

**• Accuracy **is the proportion of correctly predicted proteins. The computational formula is Eq. (4).

(2) $$\text{Sensitivity = }TP/)\left(TP+FN\right)$$

(3) $$\text{Specificity}=TN/\left(TN+FP\right)$$

(4) $$\text{Accuracy = }\left(TP+TN\right)/)\left(TP+FP+TN+FN\right)$$

In the formulas aforementioned, TP and FN refer to true positives and false negatives where TN and FP refer to true negatives and false positives.

### Web server

The web server was built on the developing environment of LAMP, and program language *perl *[21] was used for processing operator. The detail versions of these software are: Linux (CentOS_5.5 http://www.centos.org/); Apache (httpd_2.3.8 http://httpd.apache.org/); Mysql (MySQL-5.5.7 http://dev.mysql.com/downloads/); PHP (php_5.3.3 http://www.php.net/); and perl (perl_5.12.2 http://www.perl.org/).

## Results

### Optimization of analysis parameters

**Figure 2:**
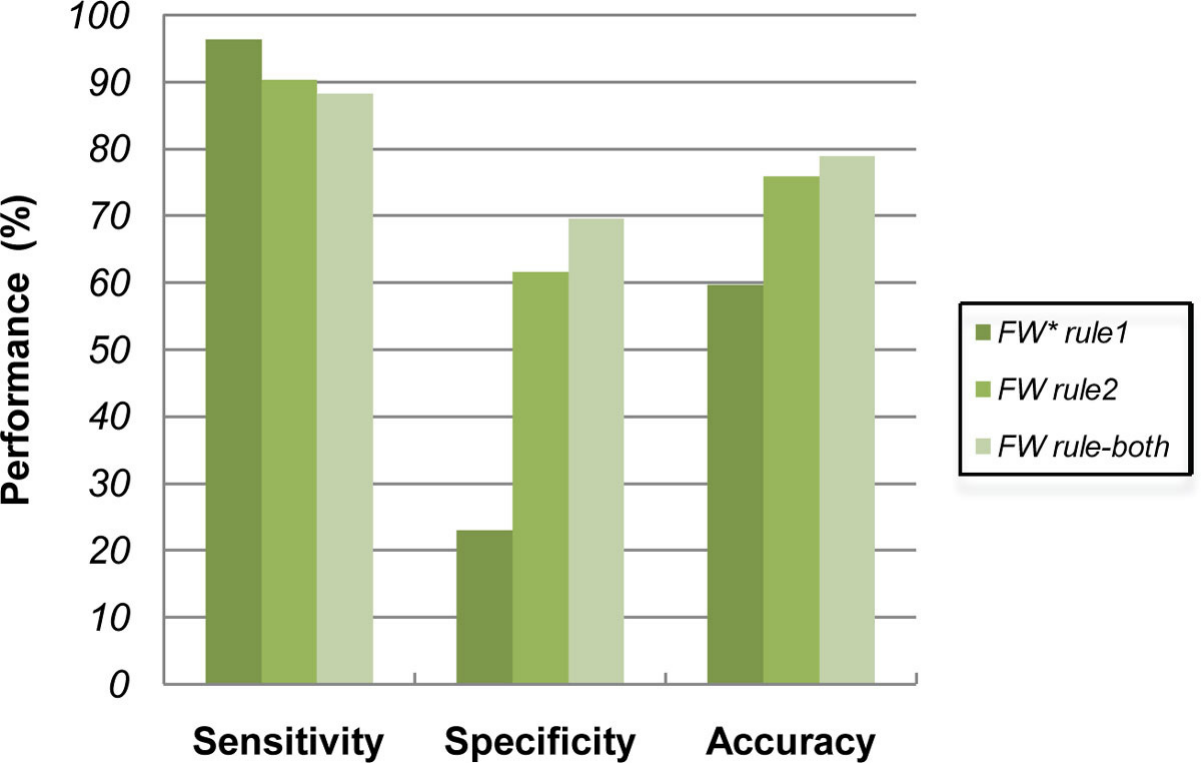
**The performance of FAO/WHO criteria**. FW* denotes FAO/WHO. The figure displayed the comparison result of each FAO/WHO criterion. Both rule 1 and rule2 had a high sensitivity, even greater than 90% with the rule 1 individually. However the corresponding specificity was only 23.05%.

We performed evaluation of the sequence-based methods meeting the criteria of FAO/WHO rule 1 (exact match a stretch of six or more consecutive identical amino acids) and rule 2 (alignment result with detections of sequence identity of 35% within any window of 80 amino acids) and rule-both respectively. As shown in Figure 2, results generated from both rule 1 and rule 2 criteria had a high sensitivity, e.g., greater than 90% using the method based on the rule 1 individually. However the corresponding specificity of this approach was only 23.05%.

**Figure 3:**
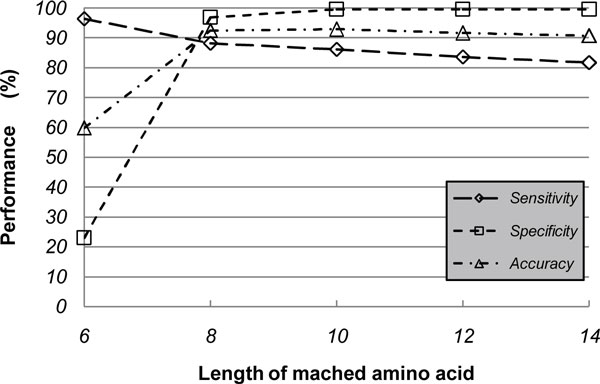
***Wordsize *influence on the capability of FAO/WHO rule 1**. The map illustrated the FAO/WHO rule 1's performance variation trend of adjusting the length of exact matched amino acids from 6 to 14. The accuracy ameliorated dramatically with increasing of *wordsize *from 6 to 8. No significant improvement was observed when we increased *wordsize *further.

**Figure 4:**
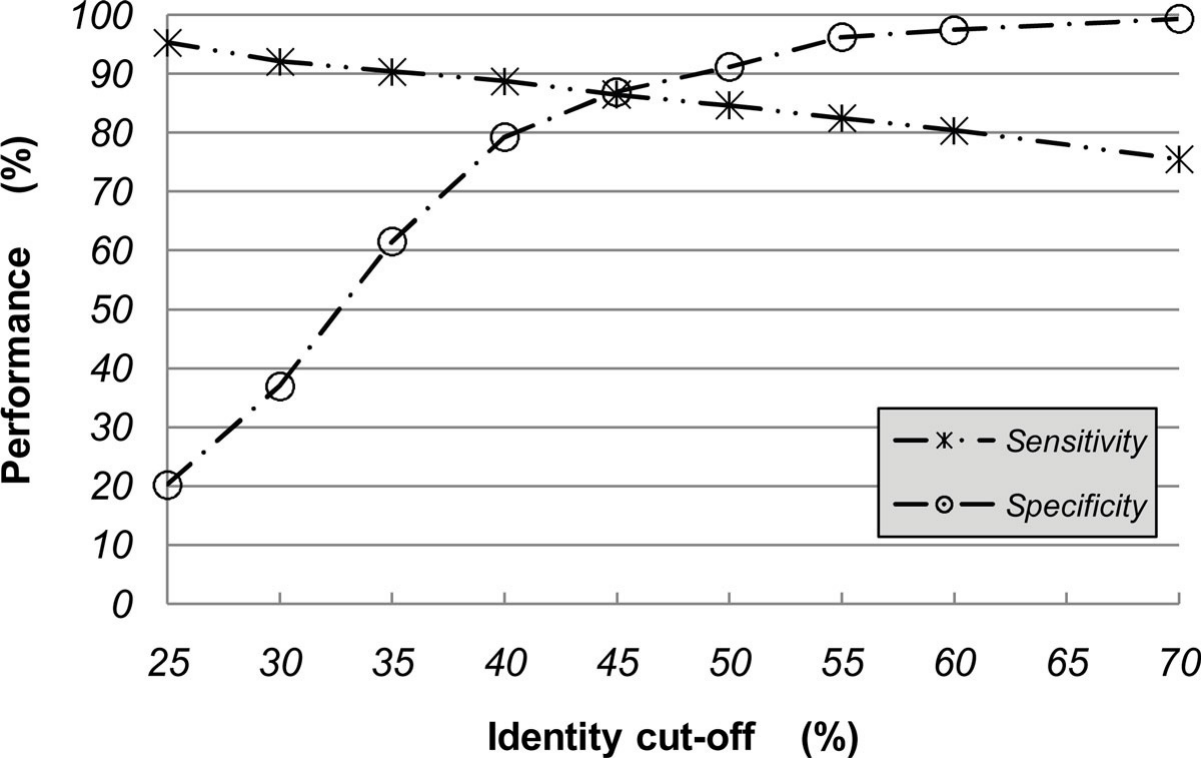
**The impact of sequence similarity on FAO/WHO rule 2**. It showed the FAO/WHO rule 2's performance by adjusting the sequence identity threshold from 25% to 70%. With the threshold increasing, the specificity rose up to 99.39% from 20.22% and the sensitivity dropped a slight. The best accuracy was obtained at identity of 55%.

To investigate the influence of *wordsize *and the identity threshold, we did the evaluation with *wordsize *from 6 to 14 step by step. As indicated in Figure 3, the accuracy ameliorated steeply as the increases of *wordsize *from 6 to 8, and in particular, the specificity increased to more than 95% from 23%. When we further increased *wordsize*, and no significant improvement was observed. In addition, the sequence identity threshold was configured from 25% to 70% gradually with an enormous rise of specificity up to 99.39% from 20.22% and a slight drop of sensitivity (Figure 4). The best accuracy was obtained at identity of 55%.

The performance of motif-based approach was summarized in Table 1. In this study, we implemented a series of E-values (0.001, 0.01, 0.1, 0.5, 0.7, 1.0 and 10.0) in MAST to investigate its effect on specificity and sensitivity. The results showed that the performances changed quite slight when MAST E-value was below 0.1 (Table 1). The sensitivity was increased from 62.63% to 66.67% while the specificity decreased from 98.99% to 96.97%. However, when the E-value was increased to more than 0.1, the specificity obviously decreased: 96.97% at 0.1, and 66.67% at 1.0. We also implemented a non-iteratively process, and found that the sensitivity dropped to less than 15% at most cases (Table 1). Considering accuracy, iteration motif elicitation with a MAST E-value of 0.5 was the suitable.

**Table 1:** Motif-based approach's performance on different MAST E-values

MAST E-value		0.001	0.01	0.1	0.5	0.7	1	10
**Iteration**	**Sensitivity**	62.63%	63.64%	66.67%	77.78%	80.81%	82.83%	100%
	**Specificity**	98.99%	98.99%	96.97%	81.82%	76.77%	66.67%	0%

**Non-iteration**	**Sensitivity**	13.66%	13.66%	13.66%	13.95%	14.16%	14.77%	23.56%
	**Specificity**	100%	100%	99.70%	99.19%	98.89%	98.48%	86.15%

### Methods comparison

**Figure 5:**
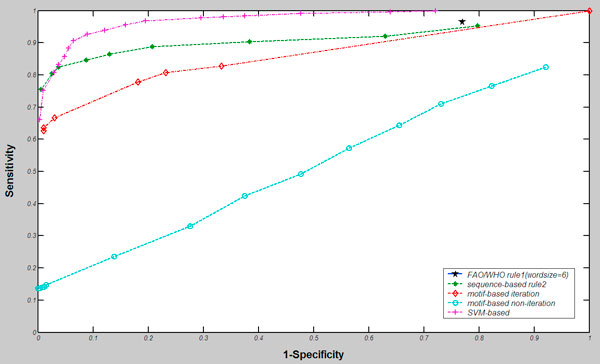
**The ROC curves of various approaches for allergen prediction**.

As described in the previous section, the amino acid composition was utilized as input vectors of dimension 20 for training and testing. As shown in Figure 5, the accuracy of 91.70% was reported with the sensitivity of 92.82% and the specificity of 90.59% (AUC = 0.97) using SVM-based approach. In addition, we compared SVM-based method with sequence- and motif-based methods. The ROC curves in Figure 5 showed that the FAO/WHO criteria had good sensitivities, but it had quite low specificity. Especially the specificity for meeting the requirement of exact matched six or more consecutive identical amino acids was only 23.05%. In other words, only less than one-fourth putative allergens were the real positive proteins using the criteria of FAO/WHO rule 1. Compared with FAO/WHO criteria, the motif-based approach had a better performance, 98.99% for specificity, 63.64% for sensitivity when MAST E-value was set as 0.01. Using the amino acid composition as feature vectors, a better result compromising the sensitivity and specificity was observed using the SVM-based method, which reached an accuracy of 91.71% while both sensitivity and specificity exceed 90%.

In addition, we did the time-consuming comparison of these prediction approaches. Table 2 listed the running time for querying one protein using each of these methods respectively. These numbers reflected that the sequence-based method needs much longer running time than those of other approaches. The SVM-based approach is most time-saving and this is very important for doing the scanning of allergen at the whole proteome level. To clarify it, we assumed the numbers of known allergens and presumed non-allergens and the length of longest sequence m,n,l respectively. To get result for one querying protein, FAO/WHO rule 1 should consume $O\left(m^{*}l^{2}\right)$ time since it need comparison of each amino acid of querying sequence to each of the sequences in known allergens database. The figure is $O\left(m^{*}F\left(m\right)\right)$ for rule 2 where $F\left(m\right)$ means the computational complex function of alignment algorithms NCBI-BLAST [27,28] or FASTA [29]. Whereas, once the motifs extraction and SVM model were finished, the computational complexity was $O\left(C\right)$ of motif-based and SVM-based approaches on prediction.

**Table 2:** Running time of each approach for querying one protein

Approaches	FAO/WHO rule 1 n* = 6	FAO/WHO rule 1 n* = 8	FAO/WHO rule2	Motif-based	SVM-based
Time (ms*)	15940	29410	58640	87	10

*n means the number of exactly matched amino acids; **ms **means millisecond

### Integrative web-based server

**Figure 6:**
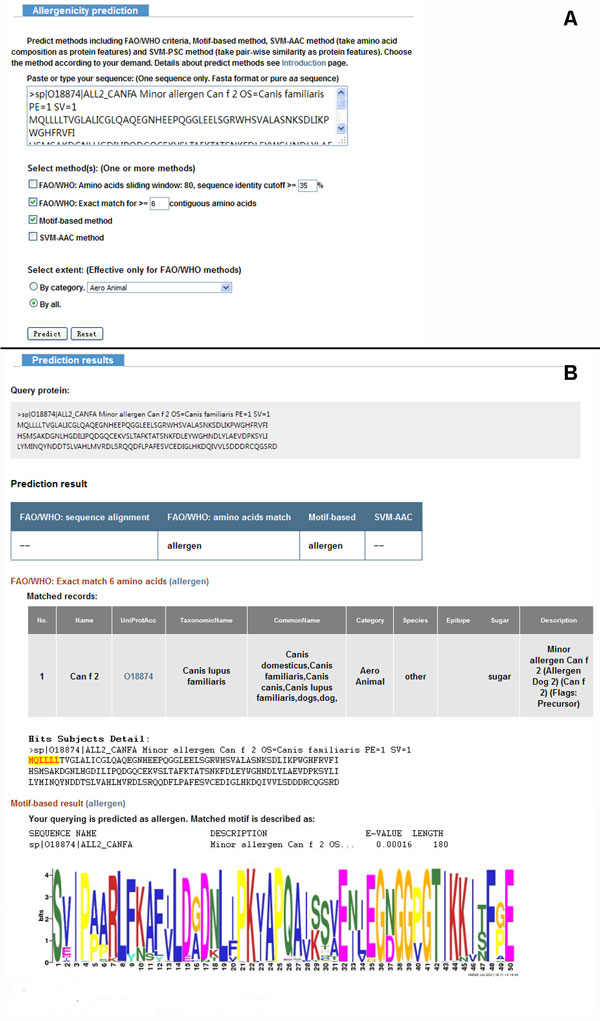
**Snapshot of web server pages**. (A) A snapshot of the sequence submission page; (B) A snapshot of the prediction result page.

Based on the computational allergen-predictive methods in this study, an integrative web application named proAP has been developed that allows user to do one-stop search for all known allergens or allergenic prediction for unknown ones using individual or combined bioinformatic methods. It allows the search of allergens by species as well as by category. According to the amount of allergens within one species, the web-based server lists top 25 species and others for user selection. As listed by Schein *et al *[19], we also divided all known allergens to 13 categories for searching such as aero animal, aero fungi, food animal, food plant, and so on. All these bioinformatic approaches investigated above were made available in proAP for allergen prediction. The user can select any of these approaches or their combination, and then a corresponding integrative result will be returned. The output page provides comprehensive information about the prediction that includes threshold, detail alignment, motif profile and probability. Especially for the approaches based on FAO/WHO criteria, the *wordsize *(the number of consecutive identical amino acids exactly matched) and the identity threshold were customizable. Both protein sequences in FASTA format and as plain text format are accepted for allergen prediction in proAP. A snapshot of the sequence submission page and prediction result page of the server is shown in Figure 6A and 6B. Beyond that, proAP also provides batch prediction, which requires users to upload protein sequences file in FASTA format and returns the results to users at the email addresses they preferred. The server and related information is available at http://gmobl.sjtu.edu.cn/proAP/main.html.

## Discussion

This study comprehensively evaluated the existing computational methods and provided a guide for predicting protein allergens. We built a uniform test dataset composed of all known allergens and putative non-allergens to evaluate mostly used computational allergen-predictive methods with ten-fold cross-validation. The comparison results showed that the SVM-based method significantly has advantages in the accuracy and processing time over the sequence-based and motif-based ones, whereas FAO/WHO criteria have a higher sensitivity and the motif-based approach may give a view on the key allergenic motif. Although a number of resources in allergen search or prediction have been reported previously, some of them provide the search of known allergen alone, such as WHO/IUIS Allergen Nomenclature [30]. And even in the other tools, only one or two computational methods of allergen prediction were available [9-11,13,15,17]. Accordingly, we built an integrative web application including the most commonly used methods and providing individual or combination allergen prediction on-line in addition to the data search of known allergens, so that users can pack individual or multiple methods in customized way according to their own purpose. Moreover, the batch prediction in proAP is very useful feature in practice that has not been implemented in any existing web tools yet.

Also, the impacts of *wordmatch *and *sliding window *in the sequence-based method were analyzed. And the performances of the motif-based approach with a variety of E-values were investigated and displayed in this study. These results are very helpful for optimizing parameters in allergen prediction. Low specificity was obtained under FAO/WHO criteria, and this situation improved significantly when we aggrandizing the number of matched amino acids or identity threshold. But it should be noticed that the computational complexity may rise accordingly when longer length of matched sequence is required. In the long term, either motif-based or SVM-based method has a "re-build" problem because one has to re-extract motifs and re-build SVM model when new allergens are detected and to be added in the positive database.

Furthermore there are several issues that could be addressed in future studies. Firstly, the existing computational methods predict allergenicity with good precision for those proteins that have high sequence similarity with the known allergens, but they are less effective when the overall similarity is low. We still can not answer clearly why a protein is more like become an allergen while the other not. Since allergenic proteins were reported have specific physiological functions and highly similar folding structures [31-34], taking the protein families classification and folding or 3D structures into the allergen prediction would be helpful to solve this issue. Secondly, more features besides protein amino acid sequence, such as biochemical characteristics and subcellular location can be included in SVM-based prediction. At last, the feature components may be sorted and selected by statistic method to optimize the performance of predictor [35,36].

## Conclusions

In summary, we systematically evaluated the performances of commonly used approaches in prediction of allergens, and developed an integrative web-based application proAP for users to more comprehensive, friendly and flexible search or predict of allergenic proteins.

## Competing interests

The authors declare that they have no competing interests.

## Authors' contributions

JW carried out the comparison of allergen-predictive methods, participated in the database setup and drafted the manuscript. YY participated in data collection and the design of the database proAP. YZ participated in parameter optimization of the methods. DZ participated in the design of the study and drafting of manuscript. JL conceived of the study, and participated in its design and coordination, and helped to draft the manuscript. All authors read and approved the final manuscript.

## Declarations

This supplement was funded by National Transgenic Plant Special Fund (2011ZX08011-006) and Program for "Chen Xing" Young Scholars, Shanghai Jiao Tong University.

This article has been published as part of *BMC Bioinformatics *Volume 14 Supplement 4, 2013: Special Issue on Computational Vaccinology. The full contents of the supplement are available online at http://www.biomedcentral.com/bmcbioinformatics/supplements/14/S4

## References

[B1] Taylor SL Protein allergenicity assessment of foods produced through agricultural biotechnology Annu Rev Pharmacal Toxical 2002 42 99 112 10.1146/annurev.pharmtox.42.082401.130208 11807166

[B2] Lee YH Sinko PJ Oral delivery of salmon calcitonin Adv Drug Deliv Rev 2000 42 225 238 10.1016/S0169-409X(00)00063-6 10963837

[B3] Mekori YA Introduction to allergic diseases Crit Rev Food Sci Nutr 1996 36 Suppl. S1 S18 8959375 10.1080/10408399609527756

[B4] Nieuwenhuizen NE Lopata AL Fighting food allergy: Current Approaches Ann N Y Acad Sci 2005 1056 30 45 10.1196/annals.1352.003 16387675

[B5] Metcalfe DD Astwood JD Townsend R Sampson HA Taylor SL Fuchs RL Assessment of the allergenic potential of foods derived from genetically engineered crop plants Crit Rev Food Sci Nutr 1996 36 Suppl. S165 S186 8959382 10.1080/10408399609527763

[B6] Codex Alimentarius Commission Joint FAO/WHO Food Standard Program Codex Alimentarius Commission 2001 Rome

[B7] FAO/WHO Evaluation of allergenicity of Genetically Modified Foods Report of a Joint FAO/WHO Expert Consultation on Allergenicity of Foods Derived from Biotechnology 2003 Rome

[B8] Ladic GS Current codex guidelines for assessment of potential protein allergenicity Food Chem Toxicol 2008 46 suppl. 10 S20 S23 18708115 10.1016/j.fct.2008.07.021

[B9] Fiers MW Kleter GA Nijland H Peijnenburg AA Nap JP van Ham RC Allermatch™, a webtool for the prediction of potential allergenicity according to current FAO/WHO Codex alimentarius guidelines BMC Bioinformatics 2004 5 133 10.1186/1471-2105-5-133 15373946 PMC522748

[B10] Zhang ZH Koh JL Zhang GL Choo KH Tammi MT Tong JC AllerTool: a web server for predicting allergenicity and allergic cross-reactivity in proteins Bioinformatics 2007 23 4 504 506 10.1093/bioinformatics/btl621 17150996

[B11] Kim C Kwon S Lee G Lee H Choi J Kim Y Hahn J A database for allergenic proteins and tools for allergenicity prediction Bioinformation 2009 3 8 344 345 Apr 21 10.6026/97320630003344 19707297 PMC2720674

[B12] Stadler MB Stadler BM Allergenicity prediction by protein sequence FASEB J 2003 17 9 1141 1143 12709401 10.1096/fj.02-1052fje

[B13] Saha S Raghava GP AlgPred: prediction of allergenic proteins and mapping of IgE epitopes Nucleic Acids Research 2006 34 W202 W209 10.1093/nar/gkl343 16844994 PMC1538830

[B14] Soeria-Atmadja D Lundell T Gustafsson MG Hammerling U Computational detection of allergenic proteins attains a new level of accuracy with *in silico *variable-length peptide extraction and machine learning Nucleic Acids Res 2006 34 3779 3793 10.1093/nar/gkl467 16977698 PMC1540723

[B15] Martinez Barrio A Soeria-Atmadja D Nistér A Gustafsson MG Hammerling U Bongcam-Rudloff E EVALLER: a web server for *in silico *assessment of potential protein allergenicity Nucleic Acida Research 2007 35 W694 W700 10.1093/nar/gkm370 PMC1933222 17537818

[B16] Cui J Han LY Li H Ung CY Tang ZQ Zheng CJ Cao ZW Chen YZ Computer prediction of allergen proteins from sequence-derived protein structural and physicochemical properties Mol Immunol 2007 44 4 514 520 10.1016/j.molimm.2006.02.010 16563508

[B17] Muh HC Tong JC Tammi MT AllerHunter: A SVM-Pairwise System for Assessment of Allergenicity and Allergic Cross-Reactivity in Proteins PLoS One 2009 4 6 e5861 10.1371/journal.pone.0005861 19516900 PMC2689655

[B18] Ivanciuc O Midoro-Horiuti T Schein CH Xie L Hilliman GR Goldblum RM Braun W The property distance index PD predicts peptides that cross-react with IgE antibodies Mol Immunol 2009 46 5 873 883 10.1016/j.molimm.2008.09.004 18950868 PMC2651743

[B19] Schein CH Ivanciuc O Braun W Maleki, SJ Structural Database of Allergenic Proteins (SDAP) Food Allergy 2006 ASM Press, Washington D.C 257 283

[B20] Smith TF Waterman MS Identification of common molecular subsequences J Mol Biol 1981 147 195 197 10.1016/0022-2836(81)90087-5 7265238

[B21] Perl 5.14.1 http://www.perl.org/

[B22] Blast-2.2.23 ftp://ftp.ncbi.nih.gov/blast/

[B23] Bailey TL Elkan C Fitting a mixture model by expectation maximization to discover motifs in biopolymers Proceedings of the Second International Conference on Intelligent Systems for Molecular Biology: 1994 1994 Menlo Park, California 28 36 7584402

[B24] Bailey TL Gribskov M Combining evidence using p-values: application to sequence homology searches Bioinformatics 1998 14 48 54 10.1093/bioinformatics/14.1.48 9520501

[B25] Chang C-C Lin C-J LIBSVM: a library for support vector machines ACM Transactions on Intelligent Systems and Technology 2011 2 27 1 27

[B26] Baldi P Brunak S Chauvin Y Andersen CA Nielsen H Assessing the accuracy of prediction algorithms for classification: an overview Bioinformatics 2000 16 412 424 10.1093/bioinformatics/16.5.412 10871264

[B27] Altschul SF Gish W Miller W Myers EW Lipman DJ Basic local alignment search tool J Mol Biol 1990 215 403 410 2231712 10.1016/S0022-2836(05)80360-2

[B28] Altschul SF Madden TL Schäffer AA Zhang J Zhang Z Miller W Lipman DJ Gapped BLAST and PSI-BLAST: a new generation of protein database search programs Nucleic Acids Res 1997 25 3389 3402 10.1093/nar/25.17.3389 9254694 PMC146917

[B29] Pearson WR Lipman DJ Improved tools for biological sequence comparison Proceedings of National Academy of Sciences of the United States of America 1988 85 8 2444 2448 10.1073/pnas.85.8.2444 PMC280013 3162770

[B30] Marsh DG Goodfriend L King TP Lowenstein H Platts-Mills TA Allergen nomenclature Bull World Health Organ 1986 64 767 74 3492310 PMC2490960

[B31] Hoffmann-Sommergruber K Pathogenesis-related (PR)-proteins identified as allergens Biochem Soc Trans 2002 30 Pt 6 930 935 12440949 10.1042/bst0300930

[B32] Ledesma A Villalba M Rodriguez R Cloning, expression and characterization of a novel four EF-hand Ca(2+)-binding protein from olive pollen with allergenic activity FEBS Lett 2000 466 1 192 196 10.1016/S0014-5793(99)01790-1 10648840

[B33] Riascos JJ Weissinger AK Weissinger SM Burks AW Hypoallergenic legume crops and food allergy: factors affecting feasibility and risk J Agric Food Chem 2010 58 1 20 27 10.1021/jf902526y 19921800

[B34] Breiteneder H Mills EN Molecular properties of food allergens J Allergy Clin Immunol 2005 115 1 14 23 quiz 24 10.1016/j.jaci.2004.10.022 15637541

[B35] Peng H Long F Ding C Feature selection based on mutual information: criteria of max-dependency, max-relevance, and min-redundancy IEEE Trans Pattern Anal Mach Intell 2005 27 1226 1238 16119262 10.1109/TPAMI.2005.159

[B36] Huang T Shi XH Wang P He Z Feng KY Hu L Kong X Li YX Cai YD Chou KC Analysis and prediction of the metabolic stability of proteins based on their sequential features, subcellular locations and interaction networks PLoS One 2010 5 6 e10972 10.1371/journal.pone.0010972 20532046 PMC2881046

